# Accelerated telomere shortening in peripheral blood lymphocytes after occupational polychlorinated biphenyls exposure

**DOI:** 10.1007/s00204-016-1725-8

**Published:** 2016-05-05

**Authors:** Susanne Ziegler, Thomas Schettgen, Fabian Beier, Stefan Wilop, Natalia Quinete, Andre Esser, Behzad Kharabi Masouleh, Monica S. V. Ferreira, Lucia Vankann, Peter Uciechowski, Lothar Rink, Thomas Kraus, Tim H. Brümmendorf, Patrick Ziegler

**Affiliations:** 1 Department of Hematology, Oncology, Hemostaseology, and Stem Cell Transplantation, Medical Faculty, RWTH Aachen University, Aachen, Germany; 2 Institute for Occupational and Social Medicine, University Hospital of the RWTH Aachen, RWTH Aachen University, Aachen, Germany; 3 Institute of Immunology, RWTH Aachen University, Aachen, Germany

**Keywords:** Polychlorinated biphenyls, Telomere, Lymphocytes, Occupational exposure

## Abstract

Polychlorinated biphenyls (PCBs) are organochlorine pollutants with a worldwide dissemination. We examined telomere length (TL) in peripheral blood cells of 207 individuals with a high body burden of PCBs due to occupational exposure in a transformer recycling company. Whereas TL in granulocytes was not affected, the age-adjusted TL in lymphocytes (∆TL_Lymph_) of exposed individuals was significantly shorter than expected [−0.77 kb; 95 % confidence interval (CI) −0.9316; −0.6052; *p* = 0.0001]. PCB exposure did not affect lymphocyte numbers or T cell receptor excision circle (TREC) levels in T cells, suggesting that PCBs cause loss of telomeric DNA in T cells due to their metabolic activation and antigen-stimulated proliferation. In support of this hypothesis, blood plasma levels of PCB-exposed individuals inhibited expression of telomerase, the telomere elongating enzyme in vitro in antigen-specific T cell proliferation assays. 3-OH-CB28, a downstream metabolite of the lower chlorinated PCB-28 in PCB-exposed individuals (mean blood plasma concentration: 0.185 ± 0.68 ng/mL), inhibited telomerase gene expression within 48 h of incubation in lymphoproliferative assays starting at a concentration of 0.27–6.75 µg/mL and accelerated telomere shortening in long-term cell culture experiments. Accelerated telomere shortening due to PCB exposure may lead to limitations of cell renewal and clonal expansion of lymphocyte populations. As PCB-related immune dysfunctions have been linked to increased susceptibility to infectious diseases and increased risk of cancer, our data provide a possible explanation, for how PCBs could promote infections and cancer through limiting immune surveillance.

**Electronic supplementary material:**

The online version of this article (doi:10.1007/s00204-016-1725-8) contains supplementary material, which is available to authorized users.

## Introduction

Polychlorinated biphenyls (PCBs) are technical mixtures consisting of up to 150 different PCB congeners (out of 209 possible congeners) which differ in position and number of chlorine atoms on two benzene rings. The general formula of PCBs is C12H10-*x*Cl*x*, where *x* represents values from 1 to 10 (World Health Organization 1976). Technical PCBs have been widely used in the last century as industrial fluids, including dielectric coolants in capacitors and transformers (Fishbein 1974; Kimbrough 1987). Although their production has been banned by industrialized countries, PCBs continue to persist as organochlorine pollutants on a global scale (Ueno et al. 2003; Choi et al. 2008; Kalantzi et al. 2001). PCBs accumulate and distribute in the human body, depending on physicochemical characteristics of individual congeners. In human blood, PCBs are enriched in the high- and low/very low-density lipoprotein fraction (Becker and Gamble 1982; Ljunggren et al. 2014) and their hydroxylated metabolites are capable of binding to lipoprotein-associated transthyretin (Purkey et al. 2004; Lans et al. 1993). Blood plasma of the general population mostly contains detectable levels of the more persistent higher chlorinated PCBs, for which diet is the main cause of uptake (Kimbrough 1987). In addition, accidental and occupational exposure of human individuals to technical PCB-mixtures has been reported (World Health Organization 1976; Chen et al. 1980; Kraus et al. 2012; Kuratsune et al. 1972). Based on epidemiological studies and animal experimentation, the International Agency for Research on Cancer (IARC) classified PCBs as human carcinogens (Lauby-Secretan et al. 2013). Furthermore, various non-carcinogenic effects, including immunologic dysfunctions, have been linked to PCB exposure in several epidemiological studies (Weisglas-Kuperus et al. 2000; Heilmann et al. 2006).

Telomeres are highly repetitive nucleotide sequences at the end of chromosomes. They protect chromosomes from erosion and fusion and are important for maintaining genomic stability (Blasco 2005). In normal somatic tissues, telomeres decrease with aging in vitro and in vivo and therefore reflect the proliferative history of somatic cells. Critically shortened telomeres have been associated with replicative exhaustion and tissue failure (de Lange 1998). Several tissues, including germline, embryonic or adult stem cells, are able to protect themselves against telomere shortening by expressing telomerase (*htert*), the telomere-extending enzyme (Flores et al. 2006). Furthermore, activated T cells respond to antigenic stimuli with clonal proliferation, thereby equally limiting telomere attrition by upregulating *htert* expression (Hiyama et al. 1995). The ability of T cells to reactivate telomerase declines after each round of stimulation, and telomerase expression levels are increasingly insufficient to maintain TL (Roth et al. 2003). Telomerase expression levels are therefore believed to impact on the lifespan of T cells (Roth et al. 2003). Several studies investigating telomere dynamics in PCB-exposed individuals or PCB-treated telomerase positive tumor cell lines have been published (Xin et al. 2016; Jacobus et al. 2008; Shin et al. 2010; Senthilkumar et al. 2011, 2012; Mitro et al. 2015). Whereas in two population-based studies low dose exposure to non-ortho PCBs was associated with longer TL in leukocytes (Shin et al. 2010; Mitro et al. 2015), long-term treatment of cell lines with PCBs resulted in cell-type and PCB congener-specific adverse effects on TL, *htert* activity and expression of telomere-associated shelterin genes (Xin et al. 2016; Jacobus et al. 2008; Senthilkumar et al. 2011, 2012). Based on these recent findings, we selectively analyzed TL of granulocytes and lymphocytes in peripheral blood from individuals occupationally exposed to very high levels of PCBs. Furthermore, we investigated the effects of plasma samples of PCB-exposed individuals on telomerase expression in proliferating, primary blood lymphocytes and characterized 3-OH-CB28, a downstream metabolite of PCB-28 in PCB-exposed individuals as a potential triggering agent of telomere dynamics in lymphocytes of individuals contaminated with a high dose of lower chlorinated PCBs.

## Materials and methods

### Participants

Participants of the present study were initially included in the medical surveillance program HELPcB (Health Effects in High-Level Exposure to PCB), which was initiated by a German Statutory Accident Insurance and a district council. The program started in 2010 after human biomonitoring revealed increased blood levels of PCB in workers of a capacitor and transformer recycling company, their relatives and workers of surrounding companies (Kraus et al. 2012). Overall, 294 adults met the entry requirements of increased PCB blood levels in the HELPcB program, as reported by Kraus et al. Due to the onset of the hematological part of HELPcB in 2011 and due to required logistical preparations, 207 collected blood samples were included in the present analysis. In total, 17.4 % of the 207 samples were donated by female and 82.6 % by male participants. Overall, 184 (88.9 %) were workers or former workers of the recycling plant or surrounding companies. In total, 20 (9.7 %) participants were relatives of these workers and 3 (1.4 %) were residents. Details of the age distribution are shown in supplementary table 1. Details of the internal PCB burden for each congener and the sum of non-dioxin-like PCB are shown in supplementary table 2a and 2b (online resource 1). The surveillance program was approved by the local ethics committee of the Medical Faculty of the Rheinisch-Westfälische Technische Hochschule (RWTH) Aachen University, Germany (EK 176/11). Participation in the program was completely voluntary, and participants could leave HELPcB any time without consequences for their insurance claim.

### Flow cytometry-based fluorescence in situ hybridization (flow-FISH)

In total, 207 peripheral blood samples of PCB-exposed individuals were processed within 48 h upon receipt. Flow-FISH was carried out according to previously described protocols (Rufer et al. 1999, 1998; Weidner et al. 2014). Briefly, samples were analyzed in triplicates with and without FITC-(C3TA2) PNA (Panagene, South Korea). Cow thymocytes with a previously determined telomere length were used both as an internal control and as a standard to translate telomere fluorescence into kb. Cow thymocytes, granulocytes and lymphocytes were identified based on forward scatter properties and LDS 751 fluorescence. TL was determined in absolute values and in relation to the respective calculated age expressed as age-adjusted telomere length (∆Tel). Blood of 104 unexposed individuals was used for age adaption as described previously (Weidner et al. 2014). Peripheral blood counts were determined using a Micros 600 hematology analyzer (Horiba ABX inc., USA).

### Statistical analysis

Significance of differences was analyzed with an ungrouped or grouped two-tailed Student *t* test. We used Spearman’s correlation coefficient for not normally distributed continuous response variables. *p* values below 0.05 were considered as statistically significant. GraphPad Prism 5.0 (GraphPad, USA) was used for statistical analysis. Additional methods are given in supplementary material.

## Results

### Telomere length analysis of peripheral blood lymphocytes derived from individuals occupationally exposed to high levels of PCBs

**Fig. 1:**
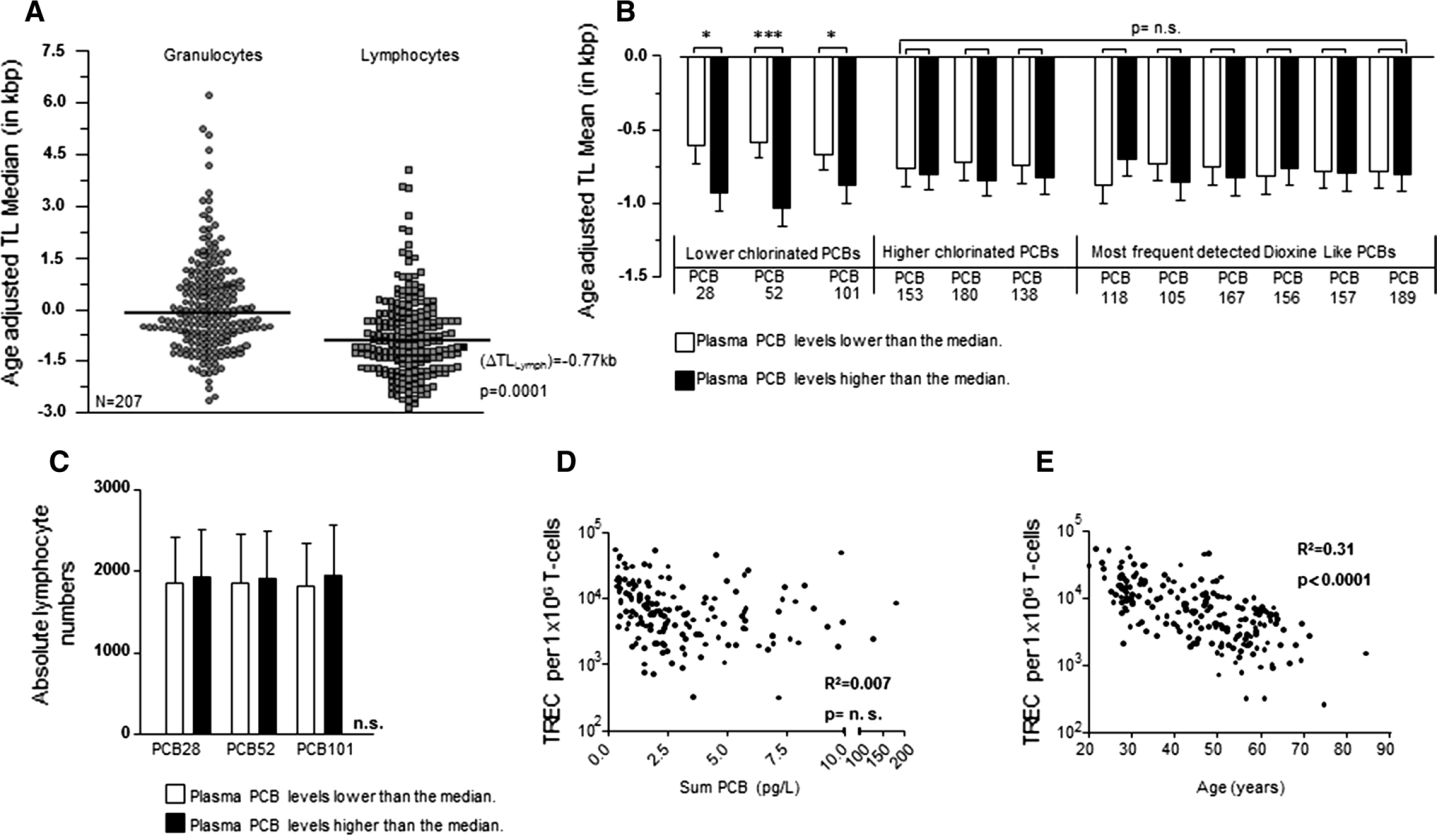
**a** Median loss of TL in the peripheral blood of PCB-exposed individuals measured by flow-FISH. TL is expressed as age-adjusted telomere length (∆Tel) and was calculated based on the TL of 104 unexposed individuals reported on previously. **b** Mean ± SD ∆Tel of lymphocytes for individual indicator PCBs depending on plasma concentration. Lower and higher concentrated plasma PCB levels were separated using the median value as cutoff. **c** Total lymphocyte numbers as determined by automated blood counting according to plasma levels of lower chlorinated PCBs. Lower and higher concentrated plasma PCB levels were split at the median. **d** Correlation between T cell receptor excision circle (TREC) levels in peripheral blood CD3+ T cells and the sum of PCBs (including lower chlorinated, higher chlorinated and dioxin-like PCBs). **e** Negative correlation between TREC levels and age of exposed individuals. Statistically significant differences are indicated (*p < 0.05; ***p < 0.001)

We compared TL in peripheral blood (PB) granulocytes and lymphocytes from PCB-exposed individuals with the TL of a population including 104 healthy controls reported on previously (Rufer et al. 1999; Weidner et al. 2014). As shown in Fig. 1a, the age-adjusted TL in lymphocytes (∆TL_Lymph_) in individuals exposed to PCBs was significantly shorter than expected (−0.77 kb; *p* = 0.0001). In contrast, the age-adjusted TL in granulocytes (∆TL_Gran_) from samples of the same cohort of PCB-exposed individuals did not differ significantly from controls (Fig. 1a).

To further analyze telomere shortening in PCB-exposed individuals, we analyzed ∆TL_Lymph_ in dependence of plasma concentrations of lower chlorinated, higher chlorinated and dioxin-like PCBs (Fig. 1b). While all cohorts had overall accelerated telomere shortening compared to age-adjusted controls, telomere shorting was more accelerated in higher contaminated individuals when compared to lower contaminated individuals. However, when broken down into individual PCB levels found in individual patients, for this comparison only the difference in the lower chlorinated PCB cohort was statistically significant.

Telomere shortening occurs during somatic cell proliferation and DNA replication (Collado et al. 2007). Thus, accelerated telomere shortening could point to an increased lymphocyte turnover in PCB-contaminated individuals (Brummendorf and Balabanov 2006). We therefore compared ∆TL_Lymph_ with absolute lymphocyte numbers within the lower chlorinated PCB cohort (Fig. 1c). However, we found no significant difference between accelerated or more accelerated telomere shortening and absolute numbers of lymphocytes. Lymphocytes, which typically comprise 14–47 % of all white blood cells (wbc), include B and T cells with CD3+ T cells accounting for 70–85 % of lymphocytes in the peripheral blood. To evaluate if T cell homeostasis is disturbed in PCB-exposed individuals, we estimated thymic T cell production by measuring T cell receptor excision circle (TREC) levels in CD3+ T cells (van Zelm et al. 2011). When correlated with the total amount of PCBs in PB, no significant correlation between TREC levels and the total amount of PCBs was detected (Fig. 1d). In contrast and as expected, TREC levels in T cells negatively correlated with the age of PCB-contaminated individuals (Fig. 1e). These findings therefore suggest that significantly shortened telomeres measured in lymphocytes of PCB-contaminated individuals are not the mere result of a PCB-induced non-specifically increased T cell turnover. Instead, PCBs seem to induce loss of telomeric DNA in T cells, presumably during their activation and antigen-stimulated proliferation.

### Blood plasma of PCB-exposed individuals inhibits telomerase gene expression in proliferating T cells

**Fig. 2:**
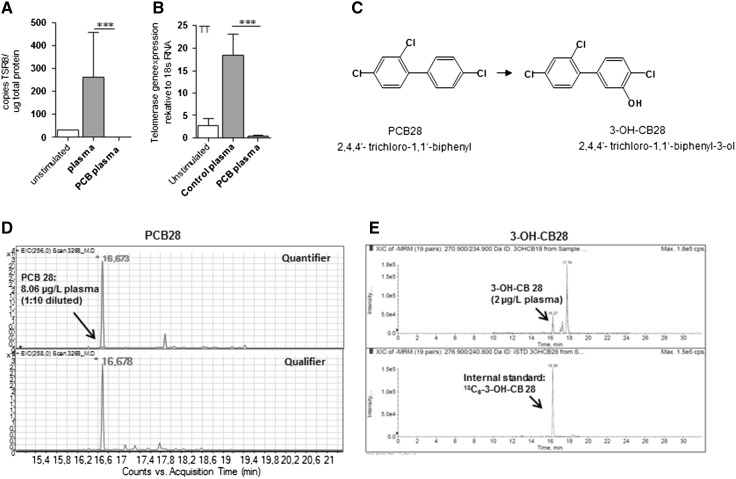
**a** PCBs inhibit telomerase activity of primary-cultured lymphocytes. Peripheral blood mononuclear cells (PBMCs) from healthy donor (blood group is 0 RhD negative) were stimulated with tetanus toxoid for 5 days, reseeded and incubated in the presence of antigen- and PCB-containing plasma samples (*n* = 9) of exposed workers or control plasma samples (*n* = 9) for 48 h. PCB-containing plasma samples were selected based on high concentrations of PCB-28. **b** Structural formula of PCB-28 and 3-OH-CB28. **c** GC/MS chromatogram of PCB-28 (*m*/*z* 256 as quantifier and 258 as qualifier) in a plasma sample of exposed workers used in A. **d** LC–MS/MS chromatogram of 3-OH-CB28 and its internal standard in the same plasma samples as used in **c**. Statistically significant differences are indicated (***p < 0.001)

T cells can be activated in vitro by specific (antigen) or unspecific (lectin) stimulation (Kruisbeek et al. 2004). After activation, T cells undergo proliferation and continued expansion, which is accompanied by the upregulation of telomerase (*tert*), the telomere elongating enzyme (Hiyama et al. 1995). To test whether PCBs in blood plasma samples of PCB-exposed individuals can modulate telomerase function in primary human T cells, we specifically stimulated peripheral blood mononuclear cells (PBMCs) with tetanus toxoid (TT) for 5 days (Fig. 2a). Subsequently, cells were reseeded and cultured in the presence of TT and medium containing either 20 % control- or PCB-contaminated plasma for 48 h. In unstimulated PBMCs, both telomerase activity and expression were very low but increased upon stimulation within a few days (online resource 1). When blood plasma of PCB-exposed individuals was added to TT-responsive T cells, both telomerase activity and expression were significantly inhibited as compared to control plasma (Fig. 2a). Thus, accelerated telomere attrition found in lymphocytes of PCB-exposed individuals in vivo could therefore be due to the active inhibition of telomerase in proliferating T cells by PCB, thereby preventing the cells from telomere maintenance upon proliferation.

To specifically test effects of lower chlorinated PCBs on telomere shortening in vitro, we focused on PCB-28 (2,4,4′-trichloro-1,1′-biphenyl, Fig. 2b), the most sensitive indicator of additional PCB contamination within this cohort of PCB-exposed individuals (Kraus et al. 2012; Schettgen et al. 2012). Similar to all lower chlorinated PCBs, PCB-28 is expected to be metabolized by the hepatic cytochrome P450 (CYP) monooxygenases enzyme system, leading to formation of hydroxylated PCB-28 (OH-CB28) (Quinete et al. 2014). However, specific information on metabolites of PCB-28 was missing. In PCB biotransformation, the phenylrings as well as the number and the positions of the chlorine atoms direct the formation of major metabolites like 4-hydroxybiphenyl as well as 3-hydroxybiphenyl and 2-hydroxybiphenyl (Groves and McClusky 1978). Based on position of unsubstituted carbon residues in PCB-28 and the theoretical mechanism of OH-PCBs formation, we concluded that 3-OH-CB28 (2,4,4′-trichloro-1,1′-biphenyl-3-ol, Fig. 2b) is a highly probable metabolite, which should be detectable in plasma of PCB-28 contaminated individuals. We therefore synthesized this putative metabolite and its labeled analogue and analyzed blood plasma levels of PCB-contaminated individuals. 3-OH-CB28 as well as the parental PCB-28 could be detected in the same plasma sample (Fig. 2c, d). However, in addition to the identified 3-OH-CB28, other unidentified peaks were present in the LC/MS/MS chromatogram, suggesting the formation of other hydroxylated metabolites of PCB-28 of unknown structure (Fig. 2d). Hence, we could conclude that blood plasma of PCB-contaminated individuals inhibits telomerase expression in a TT-specific T cell proliferation assay and that biotransformation of PCB-28 in exposed workers produces measurable amounts of OH-CB28, including 3-OH-CB28.

### 3-OH-CB28 inhibits telomerase gene expression at subtoxic concentrations in antigen-specific T cell proliferation assays

**Fig. 3:**
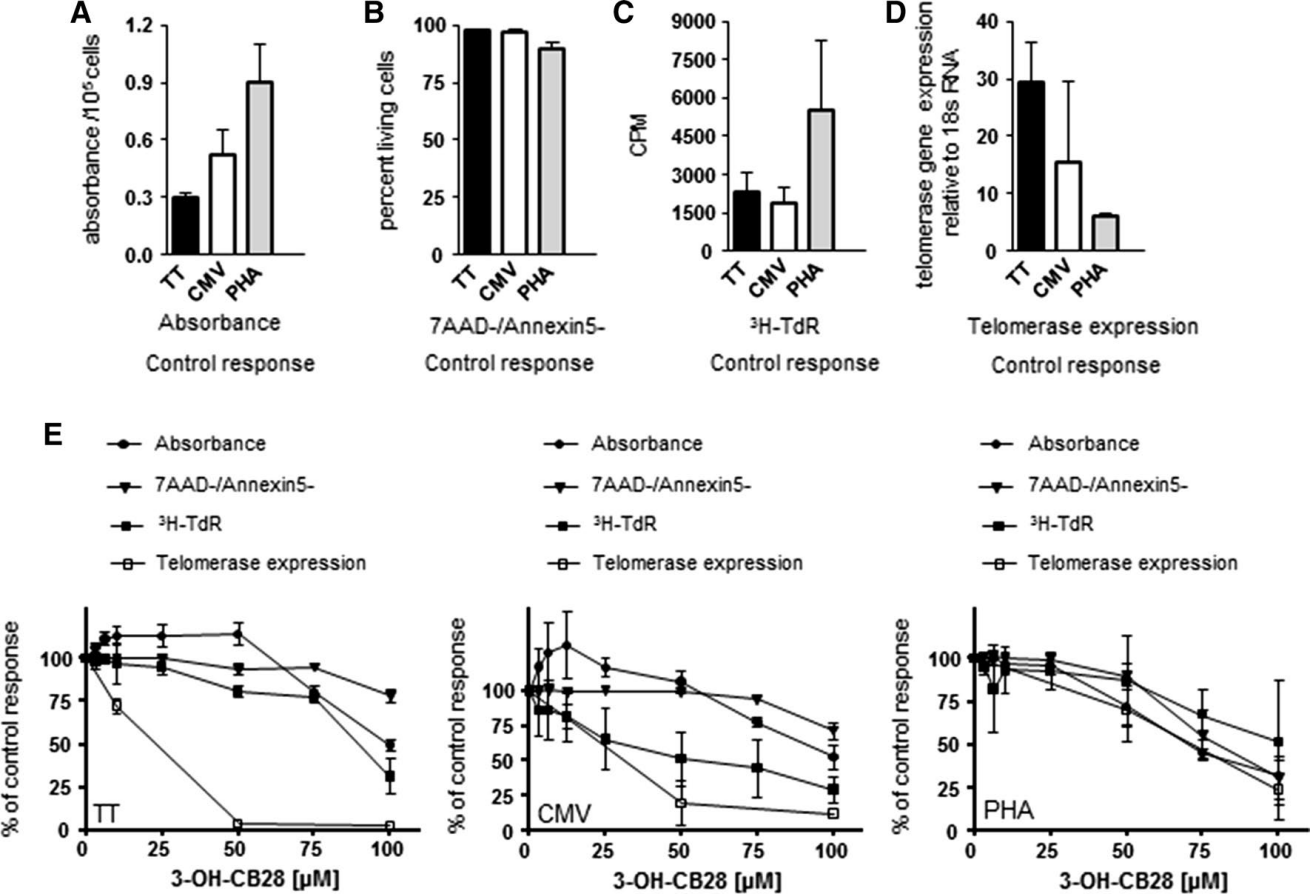
Absorbance (**a**), percentage of living cells (**b**), proliferation (**c**) and telomerase gene expression (**d**) in T cells stimulated with tetanus toxoid (TT), antigens from cytomegalovirus (CMV) infected fibroblast cell line and phytohemagglutinin (PHA). Results represent control response after a total of 7 days of stimulation without the presence of 3-OH-CB28. Mean ± SD of two different experiments with three different donors in each experiment are shown. **e** Effect of 3-OH-CB28 on absorbance, percentage of living cells, proliferation and telomerase gene expression in stimulated T cells as compared to control response, which was set to 100 %. PBMCs were stimulated with tetanus toxoid, CMV or PHA for 5 days and subsequently incubated with increasing concentrations of 3-OH-CB28 for 48 h. Cpm; counts per minute

To further characterize the effects of 3-OH-CB28 on proliferating T cells, we stimulated PBMCs with tetanus toxoid (TT) or cytomegalovirus (CMV) antigens as well as with non-specific mitogen phytohemagglutinin (PHA) (Ullman et al. 1990; Medina et al. 1994). As the TT used in this study is a single antigen, whereas the CMV proteins contain multiple antigens, a higher frequency of responding T cells in CMV- versus TT-cultured PBMCs could be expected. In PHA cultures, 95 % of T cells have been shown to respond to stimulation (Takakura et al. 2005). When added to PBMC cultures, all stimuli induced proliferation (measured as tritiated thymidine (^3^H-TdR) incorporation), high metabolic activity (given as absorbance of solubilized formazan dyes) and telomerase gene expression with low induction of apoptosis (measured as percentage of 7-amino-actinomycin negative, annexin5 negative (7AAD-/Annexin5-), living cells) within 5 days of treatment (Fig. 3a–d). Consistent with a high frequency of responding T cells, metabolic activity and proliferation were highest in cultures responding to PHA stimulation. However, when compared to TT-stimulated PBMC cultures, PHA stimulation induced only a low expression of the telomerase gene (Fig. 3a, d).

Depending on concentration, 3-OH-CB28 decreased metabolic activity as well as the proliferation of all lymphocyte cultures (Fig. 3e). In TT and CMV antigen-stimulated cultures, metabolic activity first increased by about 25 % above control levels in concentrations of 25–50 µM, but higher concentrations caused a decrease to about 50 % of control values. In PHA-stimulated cultures, no increase in metabolic activity was recognized, but at a concentration of 25 µM, metabolic activity started to decrease, reaching a level below 50 % of control response at 100 µM (all Fig. 3e). Inhibition of proliferation in TT- and PHA-stimulated cultures increased in a similar fashion with concentrations above 75 µM, necessary for a significant effect. In CMV-stimulated cultures, proliferation was inhibited at low dose (1–25 µM) and 50 µM of 3-OH-CB28 reduced proliferation by 50 %. 3-OH-CB28 did not induce apoptosis of TT- and CMV-stimulated cells at concentrations of less than 75 µM and the fraction of living cells did not fall below 75 % of control cells. In PHA-stimulated cultures, increasing numbers of dead cells were recognized as early as at 50 µM reaching levels of around 50 % of control cells.

Focusing on the telomerase gene in TT-stimulated T cells, we recognized a starting inhibition of its expression at low dose (1–25 µM), which reached almost 100 % inhibition at 50 µM of 3-OH-CB28 and was found to be independent from the inhibition of proliferation or metabolic activity as well as the induction of apoptosis (Fig. 3e; TT). In contrast, in CMV-stimulated T cells, the loss of telomerase gene expression was initially coupled to the inhibition of proliferation at low dose (1–25 µM) (Fig. 3e; CMV). Inhibition of telomerase expression after CMV stimulation reached levels below 25 % of control response at 50 µM and was more pronounced as the inhibition of proliferation. In PHA-stimulated T cells, telomerase gene expression and the metabolic activity were inhibited to a similar extend, reaching levels below 50 % of control response at 75 µM (Fig. 3e; PHA). Furthermore, in PHA-stimulated cultures, T cells reacted to increasing concentrations of 3-OH-CB28 with similar rates of decline in telomerase gene expression and proliferation as well as the number of living cells.

Taken together, we conclude that 3-OH-CB28 shows dose-dependent, significant antiproliferative and proapoptotic effects on proliferating T cells, however, with varying patterns depending on the type and specificity of T cell stimulation. Namely, in antigen driven T cell proliferation, 3-OH-CB28 inhibits the expression of telomerase, the telomere-extending enzyme, at toxic but also at subtoxic concentrations.

### Lack off effect of 3-OH-CB28 on telomerase activity in whole cell lysates

The transcriptional regulation of *htert* expression has been shown to be the primary and rate-limiting step in the activation of telomerase catalytic activity in most cell types (Takakura et al. 2005). The inhibition of *htert* expression by 3-OH-CB28 should therefore lead to a measurable inhibition of telomerase activity. In addition, a direct effect of 3-OH-CB28 on telomerase activity by interference with its catalytic subunit could have contributed to decrease telomerase activity.

**Fig. 4:**
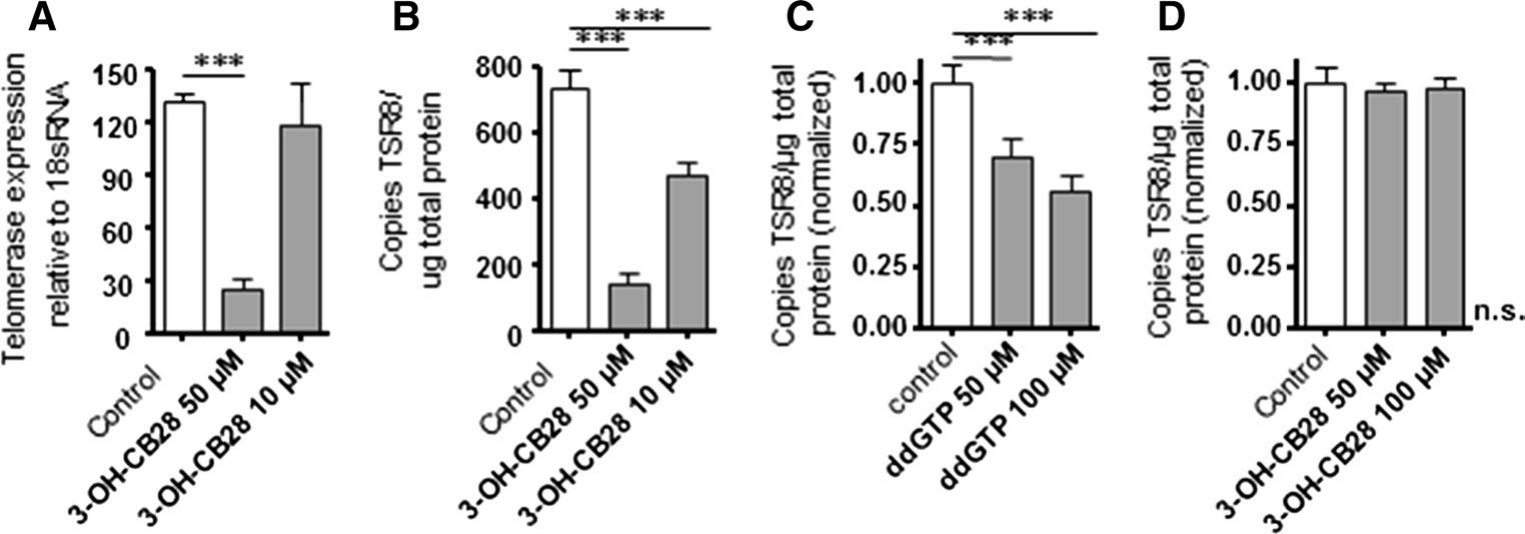
**a** Telomerase gene expression in K562 cells in the absence and presence of 3-OH-CB28. Mean ± SD of three different experiments are shown. **b** Telomerase enzyme activity in K562 cells in the absence and presence of 3-OH-CB28. Mean ± SD of three different experiments are shown. **c** Effects of the known telomerase inhibitor 2′,3′-dideoxyguanosine-5′-triphosphate (ddGTP) or 3-OH-CB28 (**d**) on telomerase enzyme activity in whole cell lysates of K562 cells. Mean ± SD of three different experiments are shown. Statistically significant differences are indicated (***p < 0.001)

To test the effects of 3-OH-CB28 on telomerase activity, we used the erythroleukemia cell line K562, which is characterized by a constitutively high expression of the telomerase gene. In K562 cells, 3-OH-CB28 inhibited telomerase gene expression in a concentration-dependent fashion and this was accompanied by a reduction in telomerase activity (Fig. 4a, b). We next compared the telomerase activity profile of whole cell lysates (WCL) from K562 cells in the presence of 3-OH-CB28 with the telomerase activity of K562 cells in the presence of the known telomerase inhibitor 2′,3′-dideoxyguanosine-5′-triphosphate (ddGTP) (Pai et al. 1998). Treatment with ddGTP reduced telomerase activity depending on concentration (Fig. 4c), while the equal concentrations of 3-OH-CB28 showed no effect on telomerase activity (Fig. 4d). We therefore conclude that 3-OH-CB28 reduces telomerase activity mainly by reducing the amount of telomerase protein, i.e., by inhibition of telomerase gene expression.

### 3-OH-CB28 accelerates telomere shortening in long-term cell culture

**Fig. 5:**
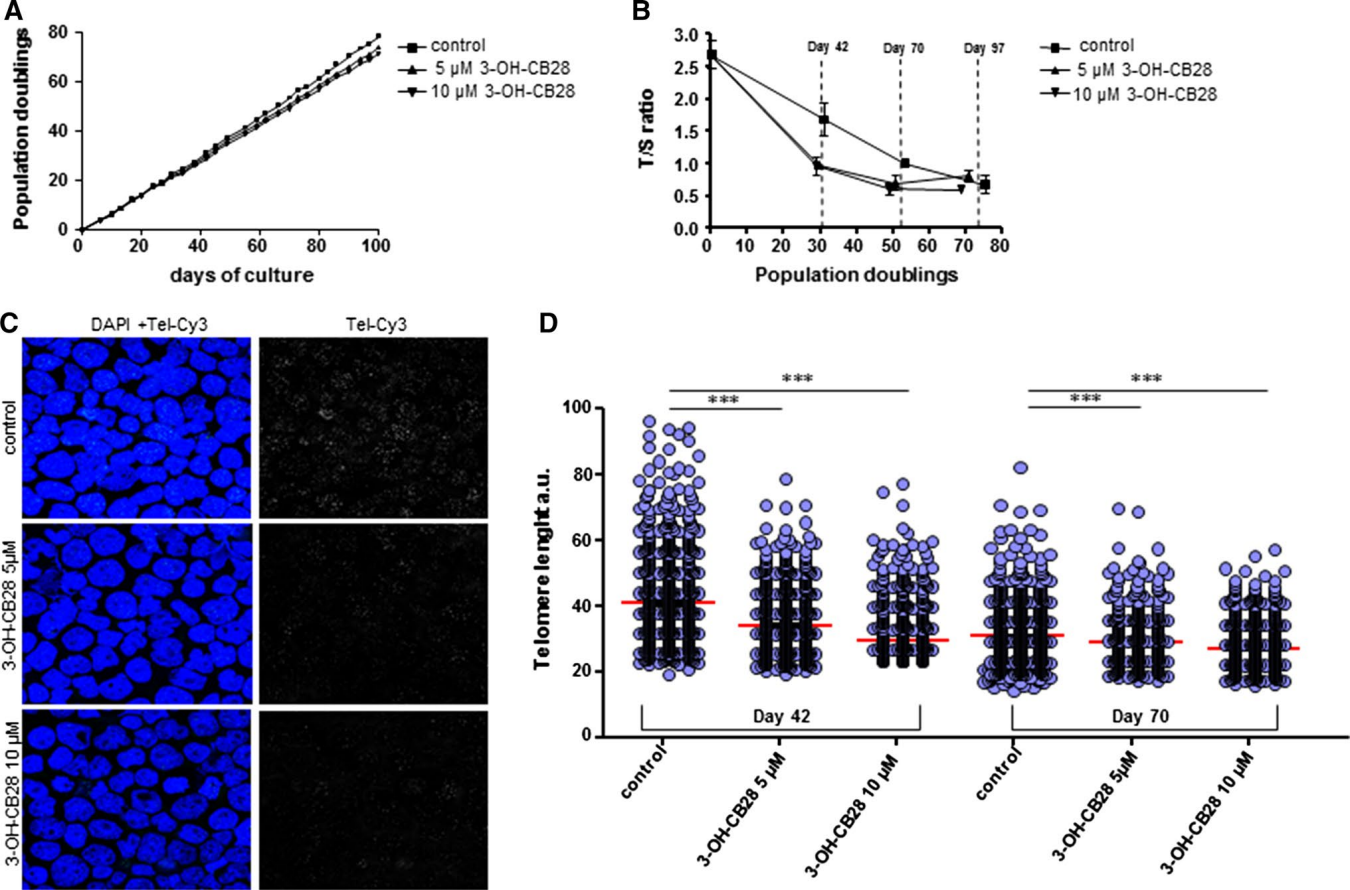
**a** Population doublings of cultured Jurkat T cells for 100 days of culture. Cells were cultured in the presence of 3-OH-CB28 at a concentration of 5 or 10 µM, or with solvent control. **b** Telomere length analysis of Jurkat T cells, cultured as described in **a** and analyzed by MM-qPCR. Relative TL is expressed as *T*/*S* ratio and plotted against population doublings. Days of culture (day 42, day 70, day 97) are indicated. **c** Representative images of cultured Jurkat T cells at day 42 of culture analyzed by confocal quantitative fluorescence in situ hybridization (confocal Q-FISH). Cells were cultured either with 3-OH-CB28 at a concentration of 5 or 10 µM, or with solvent control and subsequently stained with DAPI and a Cy3-labeled telomere probe on cytospin slides. **d** Quantification of confocal Q-FISH of Jurkat T cells by telomapping analysis for the indicated time points and culture conditions. TL quantification is given in arbitrary units of fluorescence (a.u.). Statistically significant differences are indicated (***p < 0.001)

In order to investigate the impact of 3-OH-CB28 incubation on telomere length in human T cells, we used a long-term culturing model of human Jurkat T cells which were cultured up to 80 population doublings (PDL) in vitro. Longitudinal changes in telomere length were assessed using multiplex monochrome qPCR (MM-qPCR) (Ziegler et al. 2012; Cawthon 2009) with additional assessment by confocal quantitative fluorescence in situ hybridization (confocal Q-FISH) (Varela et al. 2011) at selected time points. Up to 27 days of culture, cellular growth rate was not different between 3-OH-CB28-incubated and non-incubated Jurkat T cells. Thereafter, cell proliferation was reduced in the 3-OH-CB28-incubated cultures when compared to vehicle control (Fig. 5a). At 100 days of culture, controls reached 78 PDL, whereas 3-OH-CB28-incubated cultures only reached 73 PDL (5 µM concentration) and 71 PDL (10 µM concentration), respectively. When changes of TL at different time points during serial passaging of cells were monitored, all cultures showed telomere shortening (Fig. 5b). However, telomere shortening was more accelerated in 3-OH-CB28 incubated as compared to control cultures (Fig. 5b): Within the first 42 days of culture, we found 1.19 % change in TL per PDL in control cultures, whereas in 3-OH-CB28-incubated cultures the rate of telomere shortening was more pronounced (5 µM 3-OH-CB28: 2.13 % change per PDL and 10 µM 3-OH-CB28: 2.21 % change per PDL). Within the following 28 days of culture, changes in TL per PDL in control cultures remained at a stable rate (control: 1.14 % change per PDL) but were less in 3-OH-CB28-incubated cultures (5 µM 3-OH-CB28: 0.5 % change per PDL and 10 µM 3-OH-CB28: 0.76 % change per PDL) when compared with the first 42 days of culture. Therefore, the difference in mean TL between control and 3-OH-CB28 cultures was most pronounced and statistical significant at day 42 of culture (control *T*/*S* = 1.72 ± 0.17; 5 µM 3-OH-CB28 *T*/*S* = 1.02 ± 0.071, *p* = 0.02; 10 µM 3-OH-CB28 *T*/*S* = 0.97 ± 0.14, *p* = 0.03).

To further test effects of 3-OH-CB28 on telomere shortening of Jurkat T cells, we analyzed TL by confocal Q-FISH on cytospin-plated interphase cells. At day 42 and 70 of culture, cells with longest telomeres were enriched in controls (Fig. 5c, d). In control cultures, mean TL per nucleus was 41.8 arbitrary units (a.u.) at day 42 which decreased to 31.8 a.u. at day 70. In contrast, the mean TL in 3-OH-CB28-incubated cultures decreased from 35.7 a.u. (5 µM) and 34.8 a.u. (10 µM) at day 42 to 32.5 a.u. (5 µM) and 28.6 a.u. (10 µM) at day 70 (Fig. 5c, d). In summary, when comparing control with 3-OH-CB28-incubated cultures, Fig. 5b+d shows a similar decrease in TL using MM-qPCR and confocal Q-FISH on Jurkat T cells.

## Discussion

In this study, we describe accelerated telomere shortening in peripheral blood lymphocytes of workers occupationally exposed to high levels of PCBs. Our data suggest that exposure to PCBs causes premature telomere loss in peripheral blood lymphocytes. First, we found that the rate of telomere shortening in lymphocytes of PCB-exposed individuals depends both on the PCB congener profile and on the concentration of PCBs found in peripheral blood plasma of individuals. A high plasma concentration of lower chlorinated PCBs led to more accelerated telomere shortening than a low concentration. In addition, when used in a lymphoproliferation assay, blood plasma of PCB-exposed individuals inhibits the expression of telomerase, the telomere-extending enzyme. Finally, 3-OH-CB28, a metabolite of PCB-28, detected in the blood plasma of PCB-exposed individuals, inhibits telomerase expression when used as a single agent and accelerates telomere shortening in long-term in vitro cell expansions studies. The result of the present study strongly suggest that accelerated telomere shortening found in PCB-exposed individuals reflects the interference of PCBs with the telomerase-dependent restoration of telomere length in lymphocytes after antigenic stimulation.

Our study has several important strengths. We used advantageous quantitative fluorescence in situ and flow cytometry (flow-FISH)-based methodologies to separately assess telomere length of freshly isolated lymphoid and myeloid cells. Furthermore, due to the accurate determination of plasma concentrations of five indicator congeners (28, 101, 138, 153, 180), and seven dioxin-like congeners (105, 114, 118, 156, 157, 167) within the HELPcB program, we were able to correlate plasma PCB levels of each single participant to respective TL in lymphoid cells and to T cell receptor excision circle (TREC) levels in CD3+ T cells. In addition, the application of novel, high throughput methods for the separation of relevant congeners of OH-PCBs (Quinete et al. 2015) enabled us to identify 3-OH-CB28 as a downstream metabolite of PCB-28 in blood plasma samples of exposed individuals. The chemical synthesis of the metabolite 3-OH-CB28 and its biological testing in primary lymphocyte cultures then indicated that this metabolite is involved in telomere shortening.

This study has also limitations. We here report on the impact of a very high occupational exposure to PCBs, which was stopped as soon as the contamination had been detected. Thus, our results cannot be used to draw definite conclusions on an association between TL and exposure to PCBs on a population level. Furthermore, as a time-dependent reduction of plasma levels of lower chlorinated PCBs in participants of the study can be expected, it is not clear, whether accelerated telomere shortening in PCB-exposed lymphocytes is persistent over time. Finally, most of the participants in our study were male and sex has been shown to influence the blood concentration and the congener profile of PCBs (Knutsen et al. 2011). A fact might have biased our results.

TL in PCB-exposed individuals was approximately 770 bp shorter than in aged matched controls, and telomeres have been shown to shorten at a rate of approximately 59 bp per year in lymphocytes (Rufer et al. 1999). The reduced telomere length in PCB-exposed individuals therefore would indicate that their lymphocytes would have lost on average the equivalent TL of 13 years as compared to lymphocytes from unexposed individuals. However, the average TL is subject to considerable, genetically determined variation at any given age and TL kinetics in PCB-exposed individuals may be different depending on congener profile and concentration, complicating the relationship between age and TL. Therefore, follow-up studies, including long-term monitoring of exposed individuals, are highly warranted.

Interestingly, accelerated telomere shortening was only found in lymphocytes, whereas granulocytes displayed a normal age-adjusted TL distribution in PCB-exposed individuals. Granulocytes are postmitotic cells, and it has been shown previously by us and others in normal individuals, including frequent blood donors (Rufer et al. 1999; Scheding et al. 2003), and in defined model disorders of increased hematopoietic stem cell turnover (Beier et al. 2005; Ziegler et al. 2012; Brummendorf et al. 2000, 2001), that their telomere length reflects the proliferative history of the hematopoietic stem and progenitor cells (HSPC) they are derived from (reviewed in Brummendorf and Balabanov 2006). Therefore, the observation that no accelerated telomere shortening was found in granulocytes argues against enhanced telomere attrition in the underlying HSPC compartment in the PCB-exposed individuals studied here.

Effects of lower chlorinated PCBs on TL maintenance have been described for human skin keratinocytes and involve the inhibition of the telomerase gene (Senthilkumar et al. 2011). In addition PCB126 (dioxin-like) and PCB153 (non-dioxin-like) reduced telomerase activity in HL-60 cells and shortened telomeres after 30 days of exposure (Xin et al. 2016). Our finding that blood plasma of PCB-exposed individuals inhibits telomerase expression in lymphoproliferation assays is well in line with these previous reports. Telomerase is known to counteract telomere attrition in expanding T-lymphocytes, but the ability of long-living memory T cells to reactivate telomerase declines after consecutive rounds of antigenic stimulation (Roth et al. 2003). Periodical challenge with a common pathogen, such as influenza, or persistent infections, such as CMV, leads to repetitive expansions of the recognizing memory T cell pool through the entire life of a person (Wherry 2011; Kahan et al. 2015). As a consequence, accumulation of senescent T cells is observed in the elderly, eventually leading to infections, reduced effectiveness of vaccination and higher incidences of cancer (Wikby et al. 2006). In PCB-exposed individuals, where T cells undergo several rounds of expansion presumably without restoration of TL, premature aging of memory T cells could therefore cause higher rates of immune exhaustion after shorter time of infection. In this respect, it should be noted that PCB-associated adverse effects on adaptive immunity have been reported and include reduced antibody responses in vaccination studies (Weisglas-Kuperus et al. 2000). Furthermore, short telomeres have been shown to promote a cancer prone microenvironment (Pereira and Ferreira 2013) and are associated with reduced survival after cancer (Weischer et al. 2013). Along this line, the here described accelerated telomere shortening in peripheral lymphocytes might therefore contribute to some of the known cancerogenic effects of PCBs.

Since the 1980s, numerous studies have investigated the effects of human exposure to PCB (Kimbrough 1987). However, specific knowledge of health consequences of exposures to lower chlorinated PCB is poor. Lower chlorinated PCBs are more readily biotransformed than higher chlorinated PCBs and mainly act through downstream metabolites. Hydroxylated polychlorinated biphenyls (OH-CBs) are major metabolites of PCBs and have been shown to bind to and to interfere with steroid receptors, such as estrogen receptor-α (ERα) and Erβ (Korach et al. 1988; Connor et al. 1997). Both agonistic and antagonistic activities on estrogen receptor-mediated processes are described, especially for para- and ortho-OH structures (Takeuchi et al. 2011). Ligand-activated ERs bind through estrogen-responsive elements to the *htert* promoter and modulate telomerase gene expression by direct and indirect effects (Kyo et al. 1999; Falchetti et al. 1999). Consequently, when androgens are added to lymphocytes, which are proliferating in response to antigen, htert activity increases and this is accompanied by the upregulation of *htert* expression (Calado et al. 2009). Furthermore, estrogen deficiency has been shown to lead to telomerase inhibition, telomere shortening and reduced cell proliferation in the adrenal gland of mice (Bayne et al. 2008). 3-OH-CB28 could therefore be speculated to inhibit telomerase expression and activity by interference with ERs. However, the involvement in ERs in antigen receptor-mediated induction of telomerase activity in proliferating lymphocytes has not been shown to date and no evidence for direct effects of estradiol on telomerase expression at physiological concentrations could be found (Benko et al. 2012). Therefore, the mode of action of 3-OH-CB28 on suppressing telomerase expression in proliferating lymphocytes is not yet clear. Cell cycle entry (Buchkovich and Greider 1996) is also able to induce telomerase activity in T cells, and the repression of *htert* expression in the presence of 3-OH-CB28 may be an indirect reflection of cells becoming postmitotic. While this could explain the inhibition of telomerase in PHA-stimulated cultures, where the presence of 3-OH-CB28 induces the same rate of decline in proliferation and telomerase gene expression, it fails to explain telomerase repression in TT or CMV-stimulated cultures since in these antigen-stimulated cultures, the inhibition of telomerase expression by 3-OH-CB28 precedes the inhibition of proliferation.

As a single agent, 3-OH-CB28 recapitulates the effects of blood plasma, obtained from PCB-exposed workers, on telomerase expression in proliferating lymphocytes and accelerates telomere shortening in long-term cell culture. The involvement of 3-OH-CB28 in accelerated telomere shortening of lymphocytes in PCB-exposed individuals seems therefore plausible. However, as the concentrations of 3-OH-CB28 measured in the blood of occupationally PCB-exposed individuals (mean blood plasma concentration: 0.185 ± 0.68 ng/mL) and those concentrations showing effects in vitro (starting at 0.27–6.75 µg/mL) differed several orders of magnitude, other unidentified metabolites of PCBs most likely synergize or potentiate the effects of 3-OH-CB28 on telomere shortening.

## Electronic supplementary material

Below is the link to the electronic supplementary material.

Supplementary material 1 (PDF 93 kb)
