# Supplementary material for: Accelerated telomere shortening in peripheral blood lymphocytes after occupational polychlorinated biphenyls exposure
Source: Arch Toxicol. 2016 May 5;91(1):289–300. doi: 10.1007/s00204-016-1725-8 (PMC5225163; doi:10.1007/s00204-016-1725-8)
Supplement: Supplementary file 1 — Supplementary material 1 (PDF 93 kb) [file 204_2016_1725_MOESM1_ESM.pdf]

Electronic Supplementary Material to: Accelerated telomere shortening in peripheral blood lymphocytes after occupational polychlorinated biphenyls exposure.

Susanne Ziegler[1] , Thomas Schettgen[2] , Fabian Beier[1], Stefan Wilop[1], Natalia Quinete[2], Andre Esser [2], Behzad Kharabi Masouleh[1], Monica S.V. Ferreira[1], Lucia Vankann[1], Peter Uciechowski[3], Lothar Rink[3], Thomas Kraus[2] ,Tim H. Brümmendorf[1*] and Patrick Ziegler[1,2*]

[1]Department of Hematology, Oncology, Hemostaseology, and Stem Cell Transplantation, Medical Faculty, RWTH Aachen University, Germany.

[2]Institute for Occupational and Social Medicine, RWTH Aachen University, Germany.

[3]Institute of Immunology, RWTH Aachen University, Germany

Supplemental Material and Methods:

Signal-joint T cell receptor excision circles (TREC) analysis by Real-time PCR

The determination of TREC and albumin (ALB) by real-time PCR, a method recently described by Mitchell et al. was performed with minor modifications. In brief, TREC and ALB levels were quantified using real-time PCR performed on the StepOnePlus Cycler (Life technologies, Darmstadt, Germany). DNA was isolated from 200 µl of whole blood using the Qiagen mini DNA kit (Hilden, Germany). Fast mode Real-time PCR was performed in 20 µl reaction volumes in duplicates using Select SYBR Green Master mix (Life technologies) containing 100 ng of DNA, and published primers for TREC and ALB [29]. The sizes of the 195 bp (TREC) and 206 bp (ALB) PCR products were checked by agarose gel electrophoresis. Fast mode real-time PCR conditions were as follows: 95°C for 2 min (one cycle), 95°C for 5 s, 60°C for 30 s (40 cycles). The plasmids containing human TREC and ALB were generously provided by Dr. R. Aspinall, Department of Immunology, Imperial College, London, UK. Standard curves were generated from dilutions of known concentrations of TREC and albumin plasmids. The copy number of TREC (one per cell) and ALB (two per cell) was calculated as described (Mitchell), and TREC values per 100 ng DNA could be determined. A DNA sample from a healthy donor with known copy number was included to define variance between each run and ensure comparability. The values of TREC in one million T cells per sample were obtained after counting white blood cells and determining the amounts of CD3+ T cells by FACS analysis.

Determination of PCBs and PCB-metabolites in plasma:

Determination of PCBs has been described before. In brief, 2 mL of the plasma sample was deproteinized using formic acid. The PCBs were then extracted with n-hexane, cleaned up on a silica-gel column, and analyzed by gas chromatography/electron ionization–mass spectroscopy (GC/EI-MS) in selected ion monitoring mode (SIM). For the determination of PCB-metabolites, samples (100 µL of plasma) went through an enzymatic hydrolysis to release the target compounds from plasma, followed by protein precipitation and were analyzed by an online solid phase extraction method

coupled to liquid chromatography-tandem mass spectrometry (LC-MS/MS) in multiple reactions monitoring mode (MRM). 3OH-CB28 and its labelled internal standard (^{13}C-3OH-CB28) were custom synthetized at the Max Planck Institute for Biophysical Chemistry, Facility for Synthetic Chemistry (Göttingen, Germany). For more details on the analytical method, refer to Quinete et al.

Peripheral blood mononuclear cell (PBMC) separation and lymphoproliferation assay.

Forty milliliters of peripheral blood were collected after informed consent from healthy donors. PBMCs were enriched by density gradient centrifugation at 500*g* for 45 minutes at room temperature using Percoll Hypaque. After two rounds of washing with phosphate-buffered saline (PBS), cells were taken in culture at a concentration of $1x10^7$cells per 2 mL of medium. Medium used throughout the experiments was phenol free RPMI 1640 supplemented with 2 mM glutamine, 1% (vol/vol) nonessential amino acids, 1% (vol/vol) sodium pyruvate, penicillin (50 U/mL) and streptomycin (50 µg/ml). T-cells were stimulated in the presence of IL-2 (IL-2; 40 IU per milliliter; PeproTech Inc) and either phytohemagglutinin (5 µg/mL; Sigma-Aldrich), tetanus toxoid (20 µg/mL, gift from Dr. Halex, Behring-Werke, Marburg, Germany) or CMV grade 2 antigen (Microbix Biosystems). After 5 days of incubation, cells were harvested, reseeded and cultured in the presence of the respective mitogen. For the incorporation of ^{3}H-thymidine, cells were cultured for 48 hours in the presence of 3-OH-CB28 in a 96 well plate. 18.5 kBq/well ^{3}H-thymidine (Hartmann Analytic, Braunschweig, Germany) was added for the last 20 h of incubation before harvesting. The amount of incorporated ^{3}H-thymidine as counts per minute (CPM) was assessed by a liquid scintillation beta-counter (LBK instruments, Mt Waverly, Australia).

Cytotoxicity assays

Cells were cultured in the presence of increasing concentrations (0 to 100 µM) of 3-OH-CB28 for 48 hrs. For the determination of metabolic activity, cells were plated into 96-well flat-bottomed microtiter plates (Becton Dickinson, Heidelberg, Germany) at 1×10^5 well in 100 µL of media. Cells were preincubated for 24 hours before 3-OH-CB28 was added. All analyses were performed in five

replicates. After 48 hours, viable cells in each well were assayed for their ability to convert WST-8 [2-(2-methoxy-4-nitrophenyl)-3-(4-nitrophenyl)-5-(2,4-disulfophenyl)-*2H*-tetrazolium, monosodium salt] (CCK8-assay, Dojindo Molecular Technologies) into a water soluble formazan [31]. The absorbance of the samples was measured on a Microplate Reader (Rayto, Shenzhen, China) at 450 nm. For the determination of apoptosis induction, cells were plated into 12-well flat-bottomed microtiter plates (Becton Dickinson, Heidelberg, Germany) at 9×10^5 well in 1 ml medium. Cells were cultured for 48 hours in the presence of 3-OH-CB28. After incubation, cells were stained with the Annexin V and 7-AAD Apoptosis Detection Kit (BD Biosciences, San Jose, CA) according to manufacturer's instructions. After staining, cells were washed 5 times in PBS and subsequently analyzed by flow cytometry.

Telomerase expression and activity.

Proliferating T-cells were lysed in TRIzol reagent, and RNA was purified according to the manufacturer`s instructions (Invitrogen, Carlsbad, USA). All RNA samples were subjected to DNAse I treatment. cDNA was synthesized using random hexamers primer and Superscript III reverse transcriptase according to the manufacturer`s instructions (Invitrogen, Carlsbad, CA, USA). For quantitative analysis of htert mRNA expression, 60-90 ng cDNA was analyzed for each experiment using a sequence detector (7500 Fast Real-Time PCR System; Invitrogen) and TaqMan target mixes (Assay-on-Demand Gene expression reagents; Invitrogen). For the determination of telomerase activity the TRAPeze® RT Telomerase Detection Kit (Merck Millipore, Darmstadt, Germany) was used according to the manufacturer´s instructions. Cells were lysed by repeating freeze and thaw cycels in CHAPs buffer followed by 30 minutes of incubation on ice. After centrifugation at 16 000 g for 20 min at 4°C, aliquots of the supernatant were stored at –80°C. Protein concentration of extracts was determined with the DC Protein Assay (Bio-Rad). Using the ABI Prism 7500 Fast real time cycler (Invitrogen), samples were incubated for 30 min at 30°C and amplified in 45 PCR cycles for 15 s at 94° C, 1 min at 59°C and 10 s at 45°C. The threshold cycle values (C_t) were determined from semi-log amplification plots (log increase in fluorescence versus cycle number) and compared with standard curves generated from standard telomeric repeats provided with the kit. For the determination of

telomerase activity in ddGTP or 3-OH-CB28 incubated whole cell lysates, preincubation time was extended to 120 min at 30°C. In ddGTP assays, standard curves from telomeric repeats generated in the presence of ddGTP were used.

MMqPCR

The average telomere length of Jurkat T-cells after different days of culture was estimated by monochrome multiplex Q-PCR as described. In brief, telomeres (T) and the beta-globin gene (S; single copy) were amplified on a MyIQ2 Two-color Realtime PCR Detection-System (Biorad). Analysis was performed in duplicates and serial dilutions of standard DNAs were used to calculate the T/S ratio. Amplification efficiency for the telomere and the beta-globin was between 90% and 110% in each run. Specificity of the amplification was confirmed by melt-curve analysis.

Confocal Q-FISH

Telomere Q-FISH staining was performed as described previously. Cultured cells were frozen using standard procedures and all samples were processed in parallel. After thawing, cells were spun on microscope slides and fixed with formaldehyde (Sigma, US). After three additional washing steps, slides were dehydrated with ethanol and telomeres were stained with Cy3-(C3TA2) PNA (Panagene, South Corea) followed by six further washing steps. DNA counter staining was carried out using DAPI solution (Sigma, US) and TL analysis was performed within 48 h using a high-resolution LSM710 (Zeiss, Jena, Germany) confocal microscope. Images were captured using 63x optical magnification with additional 2x zoom and multi-tracking mode on 0.5 µm steps was used to acquire images of DAPI and Cy3 staining. Maximum projection of 5 single consecutive steps was carried out and acquired images were used for further digital image analysis. TL detection was carried out using Definiens software (Definiens, Germany). Nuclei and telomeres were detected based on the respective DAPI and Cy3 intensity. Mean nuclear background of the Cy3 staining nuclei was calculated and subtracted of each detected telomere within the respective nucleus. Median value of the telomere length per single detected nucleus was used for analysis. TL quantification is given in arbitrary units of fluorescence (a.u.).

Supplemental Figures:

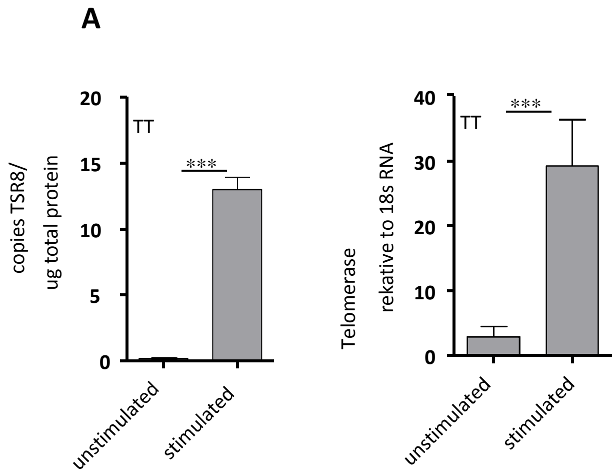

A) Telomerase activity and expression of primary unstimulated and stimulated lymphocytes. PBMCs from healthy donors were stimulated with tetanus toxoid for 5 days and telomerase activity and expression were assessed as described.

Supplemental Tables

Participants

	N	mean	median	std. deviation	minimum	maximum	25^{th}Perc.	50^{th}Perc.	75^{th}Perc.	95^{th}Perc.
Female	36	43.8	47.2	12.4	21.7	69.4	31.2	47.2	55.6	62.7
Male	171	45.9	46.4	13.1	20.1	84.3	33.0	46.4	57.0	64.1
Total	207	45.5	46.8	13.0	20.1	84.3	33.0	46.8	56.5	63.6

Table 1: Age distribution by gender

Table 2

		PCB28	PCB52	PCB101	PCB∑ lc	PCB138	PCB153	PCB180	PCB∑ ndl
N	Valid	207	207	207	207	207	207	207	207
	Missing	0	0	0	0	0	0	0	0
Mean		1.686	0.060	0.119	1.865	2.701	2.829	1.709	9.105
Median		0.062	0.005	0.019	0.093	0.738	0.987	0.806	3.015
Std. Deviation		10.287	0.226	0.322	10.640	5.884	5.687	3.103	21.226
Minimum		0.005	0.005	0.005	0.015	0.073	0.093	0.066	0.280
Maximum		121.483	2.769	2.719	126.371	61.378	59.087	31.622	179.712
Percentiles	25	0.026	0.005	0.005	0.038	0.396	0.530	0.347	1.501
	50	0.062	0.005	0.019	0.093	0.738	0.987	0.806	3.015
	75	0.361	0.026	0.071	0.556	2.224	2.579	1.663	7.656
	95	4.659	0.257	0.578	5.639	12.581	12.282	7.555	35.326

A: Distribution of non-dioxin like (ndl) PCB congeners, with Sum of lower chlorinated (lc) PCB28 + PCB52 + PCB101 and the sum of all non-dioxin like PCB

		PCB77	PCB81	PCB105	PCB114	PCB118	PCB123	PCB126	PCB156	PCB157	PCB167	PCB169	PCB189
N	Valid	207	207	207	207	207	207	207	207	207	207	207	207
	Missing	0	0	0	0	0	0	0	0	0	0	0	0
Mean		0.005	0.005	0.373	0.049	1.162	0.017	0.005	0.311	0.054	0.117	0.005	0.033
Median		0.005	0.005	0.048	0.013	0.209	0.005	0.005	0.108	0.019	0.037	0.005	0.015
Std. Deviation		0.001	0.002	1.151	0.106	3.074	0.046	0.000	0.610	0.102	0.243	0.000	0.055
Minimum		0.005	0.005	0.005	0.005	0.019	0.005	0.005	0.005	0.005	0.005	0.005	0.005
Maximum		0.016	0.029	10.349	0.809	28.852	0.479	0.005	5.589	0.866	2.387	0.005	0.482
Percentiles	25	0.005	0.005	0.018	0.005	0.081	0.005	0.005	0.054	0.010	0.016	0.005	0.005
	50	0.005	0.005	0.048	0.013	0.209	0.005	0.005	0.108	0.019	0.037	0.005	0.015
	75	0.005	0.005	0.218	0.049	0.867	0.005	0.005	0.268	0.045	0.109	0.005	0.033
	95	0.005	0.005	1.484	0.227	4.911	0.059	0.005	1.523	0.248	0.469	0.005	0.138

Table 2b: Distribution of dioxin like PCB congeners
